# Primary immunodeficiency-related genes and varicella-zoster virus reactivation syndrome: a Mendelian randomization study

**DOI:** 10.3389/fimmu.2024.1403429

**Published:** 2024-08-26

**Authors:** Hao Wang, Guanglei Chen, Qian Gong, Jing Wu, Peng Chen

**Affiliations:** ^1^ Guizhou University of Traditional Chinese Medicine, Guiyang, Guizhou, China; ^2^ Guangzhou University of Chinese Medicine, Guangzhou, Guangdong, China

**Keywords:** primary immunodeficiency-related genes, varicella-zoster virus reactivation syndrome, neurological complications, Mendelian randomization, two-sample study

## Abstract

**Background:**

Currently, evidence regarding the causal relationship between primary immunodeficiency-related genes and varicella-zoster virus reactivation syndrome is limited and inconsistent. Therefore, this study employs Mendelian randomization (MR) methodology to investigate the causal relationship between the two.

**Methods:**

This study selected 110 single-nucleotide polymorphisms (SNPs) of primary immunodeficiency-related genes as instrumental variables (IVs). Genetic associations of primary immunodeficiency-related genes were derived from recent genome-wide association studies (GWAS) data on human plasma protein levels and circulating immune cells. Data on genes associated with varicella-zoster virus reactivation syndrome were obtained from the GWAS Catalog and FINNGEN database, primarily analyzed using inverse variance weighting (IVW) and sensitivity analysis.

**Results:**

Through MR analysis, we identified 9 primary immunodeficiency-related genes causally associated with herpes zoster and its subsequent neuralgia; determined causal associations of 20 primary immunodeficiency-related genes with three vascular lesions (stroke, cerebral aneurysm, giant cell arteritis); revealed causal associations of 10 primary immunodeficiency-related genes with two ocular diseases (retinopathy, keratitis); additionally, three primary immunodeficiency-related genes each were associated with encephalitis, cranial nerve palsy, and gastrointestinal infections.

**Conclusions:**

This study discovers a certain association between primary immunodeficiency-related genes and varicella-zoster virus reactivation syndrome, yet further investigations are warranted to explore the specific mechanisms underlying these connections.

## 1 Introduction

The varicella-zoster virus (VZV) is a double-stranded DNA alpha herpesvirus, with a global infection rate that can reach up to 95% (1).VZV can lead to primary infection known as chickenpox, after which it remains latent throughout the entire neural axis, primarily residing in neurons of human neural ganglia, including the dorsal root ganglia (DRG), trigeminal ganglia (TG), and autonomic ganglia located in the enteric nervous system (2, 3). As the host’s immune function declines, VZV suppresses the expression of major histocompatibility complex class I (MHC-I), major histocompatibility complex class II (MHC-II), pattern recognition receptors, and activates T lymphocyte autophagy. This makes infected target cells difficult for the immune system to recognize, affecting the production of cytokines such as interferon (IFN). Subsequently, VZV reactivates from the ganglia and travels along nerves to the skin, resulting in herpes zoster. Herpes zoster commonly presents with postherpetic neuralgia and may also retrogradely spread, leading to a series of syndromes including stroke, encephalitis, and retinopathy (4). Although some cases of infection exhibit mild symptoms, certain patients with specific primary immunodeficiencies may progress to severe or life-threatening stages, highlighting the complexity of the pathogenesis of VZV. Exploring reliable and practical biomarkers for use in clinical prevention and treatment of VZV reactivation syndrome is of significant importance. Research indicates that certain primary immunodeficiencies predispose individuals to infection and reactivation of VZV (3, 5). For instance, defects in natural killer (NK) cells caused by mutations in genes such as FCGRIIIA and MCM4 may lead to severe or life-threatening complications associated with VZV (6). Dysfunction of plasmacytoid dendritic cells is believed to be associated with the occurrence of VZV-related retinal necrosis (7). However, the precise molecular mechanisms remain incompletely elucidated. Additionally, while numerous genes are associated with primary immunodeficiencies, the genetic relationship and potential causal associations between these genes and VZV reactivation syndrome are not yet clear.

In this context, Mendelian randomization (MR) can serve as a method to assess the causal relationship between the two. MR, a concept proposed by Katan in 1986 (8), utilizes single-nucleotide polymorphisms (SNPs) as instrumental variables (IVs) to estimate the causal effect of exposure on outcomes (9). MR can be considered as a form of natural randomized clinical trial (RCT). In contrast to typical RCT designs, the greatest advantage of MR lies in the random allocation of SNPs, serving as instrumental variables for risk factors, thereby minimizing potential confounding factors or reverse causality effects to a significant extent (10). With the accumulation of summary data from genome-wide association studies (GWAS), MR has been widely employed to infer causal associations between exposure and outcomes. In this study, two-sample MR analysis was conducted based on data from the GWAS Catalog database and FINNGEN database. The aim was to assess the causal relationship between primary immunodeficiency-related genes and VZV reactivation syndrome and to identify primary immunodeficiency gene targets associated with VZV reactivation syndrome.

## 2 Materials and methods

### 2.1 Study design

**Figure 1:**
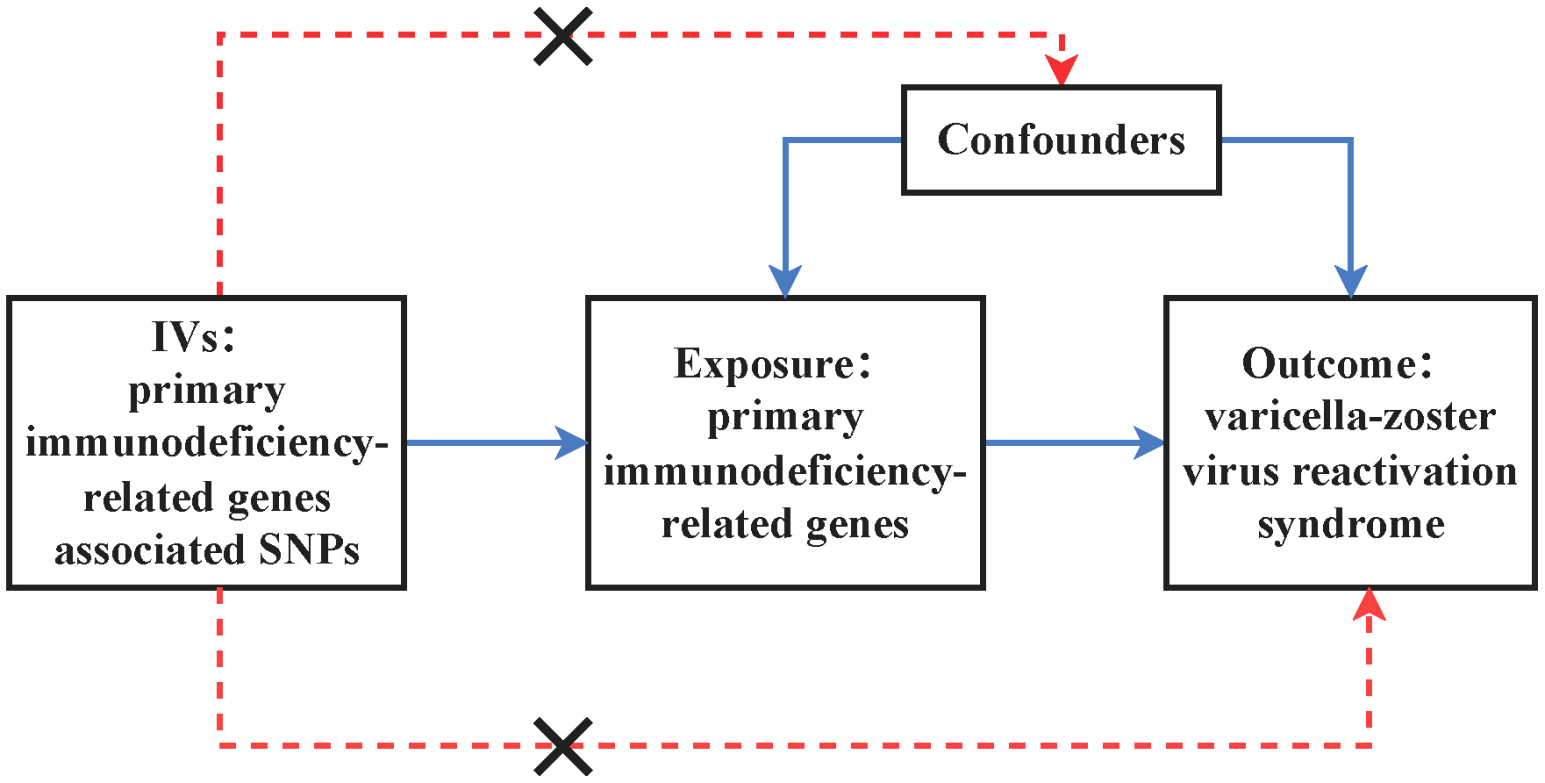
Illustrates the design of the MR study assessing the causal relationship between primary immunodeficiency-related genes and varicella-zoster virus reactivation syndrome. IVs stand for instrumental variables, while SNPs represent single-nucleotide polymorphisms.

We conducted an MR study aimed at assessing the causal relationship between primary immunodeficiency-related genes and the risk of VZV reactivation syndrome. **Figure 1** illustrates the principles of MR. Effective instrumental variables (IVs) (SNPs in this study) must satisfy the following three assumptions: (i) SNPs are strongly associated with the exposure; (ii) SNPs are independent of confounding factors that influence the association between exposure and outcome; (iii) SNPs are only associated with the outcome through the exposure (11). First, we conducted MR analysis of exposure and outcome using publicly available data from the GWAS Catalog database and the FINNGEN database, which contain summary statistics from GWAS. Since we utilized summary statistics from publicly available studies, no additional ethical approval was required.

### 2.2 Data sources

This study utilized a publicly available meta-analysis of GWAS on circulating concentrations of 110 primary immunodeficiency-related genes. The analysis was conducted on 400 unrelated healthy individuals of Western European ancestry, stratified by gender and age (20-29 years and 60-69 years), evaluating 229 proteins and 5.2 million single-nucleotide polymorphisms. It involved 152 significant associations with 49 proteins and 20 non-genetic variables, where 100 synonymous and 12 distal protein quantitative trait loci were associated with 19 new genes related to primary immunodeficiency diseases (12). We extracted summary GWAS statistics for 10 types of varicella-zoster virus reactivation syndrome from the latest data available in both the GWAS Catalog database and the FINNGEN database. Details can be found in **Table 1**.

**Table 1:** Source of all aggregated statistics.

Endpoint name in FINNGEN or GCST in GWAS Catalog	Exposure or outcome	Number of cases	Number of controls	Consortium
**GCST90085705-GCST90085814**	Primary immunodeficiency-related genes	400 individuals		GWAS Catalog
**GCST90018941**	Herpes zoster	2731	518343	GWAS Catalog
**G6_POSTZOST**	Postherpetic neuralgia	313	330377	FINNGEN
**GCST90044350**	Stroke	6986	448317	GWAS Catalog
**GCST90044003**	Cerebral aneurysm	290	456058	GWAS Catalog
**GCST90044019**	Giant cell arteritis	278	456070	GWAS Catalog
**H7_KERATITIS**	Keratitis	11611	357814	FINNGEN
**GCST90043778**	Retinopathy	605	455743	GWAS Catalog
**GCST90043730**	Encephalitis	189	456159	GWAS Catalog
**GCST90018843**	Cranial nerve palsy	2237	639537	GWAS Catalog
**AB1_INTESTINAL_INFECTIONS**	Gastrointestinal infections	40881	336396	FINNGEN

### 2.3 Selection of instrumental variables

We adopted uniform inclusion criteria for genetic variants and selected commonly used thresholds in MR analysis, with a significance threshold of *P*<5×10^-6^ for selecting significant association results. After extracting significant SNPs corresponding to each primary immunodeficiency-related gene, we performed linkage disequilibrium analysis (LD>10000kb, r^2^<0.001) using the European (EUR) genotype from the 1000 Genomes Project as a reference panel. Under these conditions, we selected 110 primary immunodeficiency-related genes. Finally, we utilized the average F-statistic to quantify the strength of SNPs (13). An average F-statistic > 10 indicates sufficient strength to ensure the effectiveness of the SNP for the trait (14).

### 2.4 Mendelian randomization analysis

We conducted MR analysis on the causal relationship between 110 primary immunodeficiency-related genes and varicella-zoster virus reactivation syndrome. MR analysis was performed using R (version 4.3.1) and the R package “Two Sample MR” (version 0.5.7) (15). The primary method used in this MR analysis was inverse variance weighting (IVW) (16), supplemented by weighted median (WM) and MR-Egger regression (MRE) methods. Since all SNPs were considered valid IVs, IVW provided reliable estimates of causal effects.

When there were at least three effective IVs, sensitivity analysis was conducted to explore potential bias caused by invalid IVs. Sensitivity analysis included tests for horizontal pleiotropy and heterogeneity. The intercept term of MR-Egger regression is commonly used to test for horizontal pleiotropy. If this intercept term significantly differs from zero, it indicates the presence of horizontal pleiotropy (*P* < 0.05) (17). Cochran’s Q test quantifies the heterogeneity of instrumental variables, where *P* < 0.05 indicates heterogeneity (18). Additionally, we employed the MR pleiotropy residual sum and outlier test (MR-PRESSO) to detect outliers (19). If outliers were detected, they were removed, and the remaining IVs were reanalyzed. The risk relationship between the 110 primary immunodeficiency-related genes and varicella-zoster virus reactivation syndrome was expressed in terms of odds ratios (ORs) and their 95% confidence intervals (CIs). Evidence for a possible causal relationship was provided if *P* < 0.05.

## 3 Results

### 3.1 Overview

**Figure 2:**
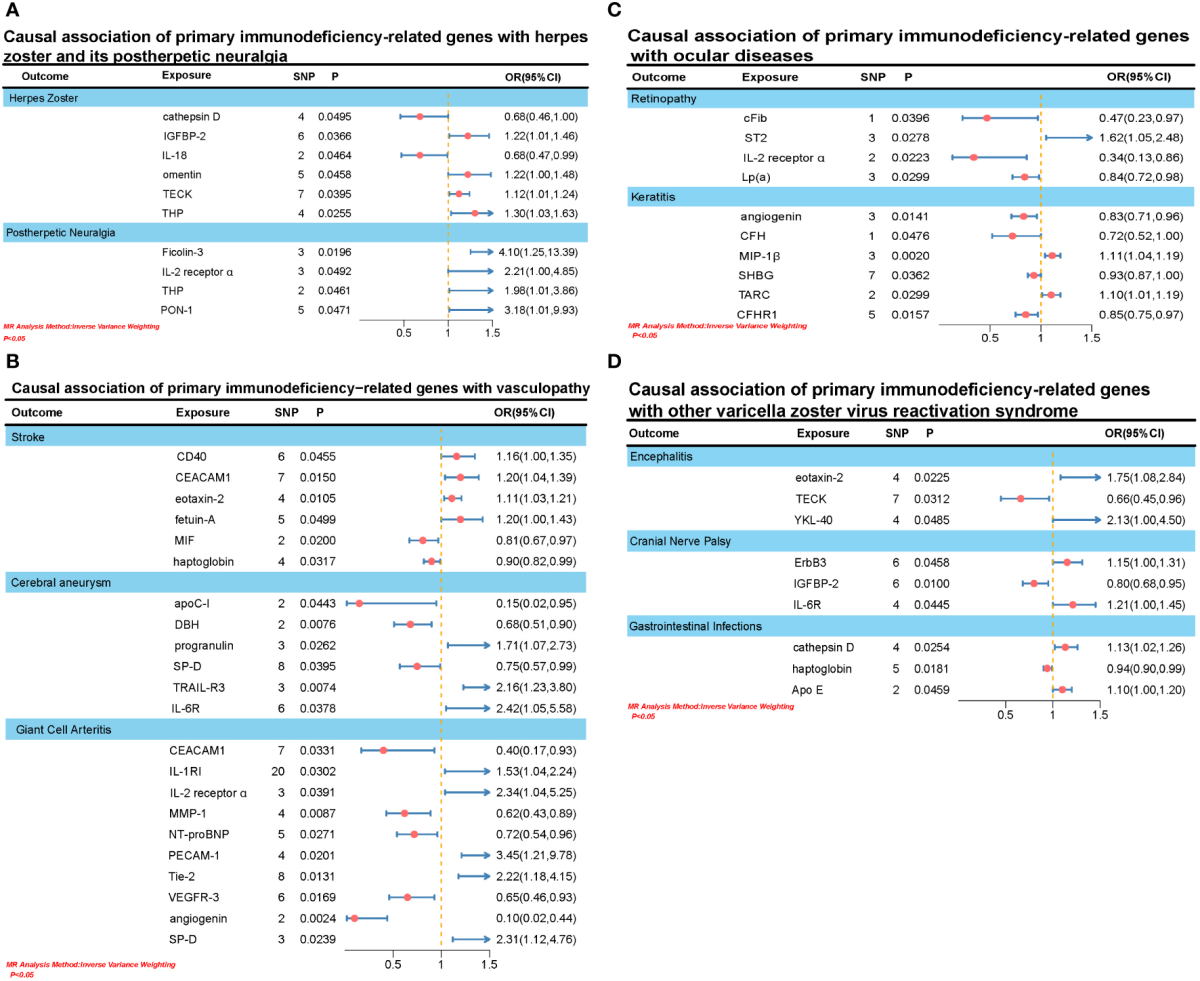
**(A)** Causal association of primary immunodeficiency-related genes with herpes zoster and its postherpetic neuralgia. **(B)** Causal association of primary immunodeficiency-related genes with vasculopathy. **(C)** Causal association of primary immunodeficiency-related genes with ocular diseases. **(D)** Causal association of primary immunodeficiency-related genes with other varicella zoster virus reactivation syndrome.

In this MR analysis, we primarily employed the IVW test method to examine the causal relationship between 110 primary immunodeficiency-related genes and varicella-zoster virus reactivation syndrome. All relationships with *P* < 0.05 in the IVW model are depicted in **Figure 2**.

### 3.2 Causal association between primary immunodeficiency-related genes and herpes zoster and its postherpetic neuralgia

We identified causal associations between six primary immunodeficiency-related genes and herpes zoster. Specifically, elevation in IGFBP-2 (OR: 1.2151, 95% CI: 1.0122-1.4587, *P_IVW_*: 0.0366), omentin (OR: 1.2176, 95% CI: 1.0037-1.4770, *P_IVW_*: 0.0458), TECK (OR: 1.1165, 95% CI: 1.0053-1.2399, *P_IVW_*: 0.0395), and THP (OR: 1.2977, 95% CI: 1.0325-1.6310, *P_IVW_*: 0.0255) was associated with an increased risk of herpes zoster. However, elevation in cathepsin D (OR: 0.6807, 95% CI: 0.4638-0.9991, *P_IVW_*: 0.0495) and IL-18 (OR: 0.6845, 95% CI: 0.4715-0.9939, *P_IVW_*: 0.0464) was associated with a decreased risk of herpes zoster. Additionally, elevation in four primary immunodeficiency-related genes was associated with an increased risk of postherpetic neuralgia, including Ficolin-3 (OR: 4.0951, 95% CI: 1.2528-13.3857, *P_IVW_*: 0.0196), IL-2 receptor α (OR: 2.2062, 95% CI: 1.0027-4.8540, *P_IVW_*: 0.0492), THP (OR: 1.9765, 95% CI: 1.0199-3.8607, *P_IVW_*: 0.0461), and PON-1 (OR: 3.1751, 95% CI: 1.0147-9.9347,*P_IVW_*:0.0471).

### 3.3 Causal association between primary immunodeficiency-related genes and vasculopathy

We identified a causal association between 20 primary immunodeficiency-related genes and three types of vascular lesions (stroke, cerebral aneurysm, and giant cell arteritis). Firstly, elevated levels of IGFBP-2 (OR: 1.1629, 95% CI: 1.0030-1.3483, *P_IVW_*: 0.0455), CEACAM1 (OR: 1.1979, 95% CI: 1.0357-1.3855, *P_IVW_*: 0.0150), eotaxin-2 (OR: 1.1119, 95% CI: 1.0251-1.2060, *P_IVW_*: 0.0105), and fetuin-A (OR: 1.1960, 95% CI: 1.0001-1.4304, *P_IVW_*: 0.0499) were associated with an increased risk of stroke. Conversely, elevation in MIF (OR: 0.8078, 95% CI: 0.6748-0.9670, *P_IVW_*: 0.0200) and haptoglobin (OR: 0.9034, 95% CI: 0.8234-0.9911, *P_IVW_*: 0.0317) was associated with a decreased risk of stroke. Secondly, elevation in progranulin (OR: 1.7069, 95% CI: 1.0654-2.7345, *P_IVW_*: 0.0262), TRAIL-R3 (OR: 2.1605, 95% CI: 1.2297-3.7959, *P_IVW_*: 0.0074), and IL-6R (OR: 2.4218, 95% CI: 1.0512-5.5795, *P_IVW_*: 0.0378) was associated with an increased risk of cerebral aneurysm. Conversely, elevation in apoC-I (OR: 0.1501, 95% CI: 0.0237-0.9526, *P_IVW_*: 0.0433), DBH (OR: 0.6810, 95% CI: 0.5136-0.9030, *P_IVW_*: 0.0076), and SP-D (OR: 0.7514, 95% CI: 0.5724-0.9864, *P_IVW_*: 0.0395) was associated with a decreased risk of cerebral aneurysm. Lastly, elevation in IL-1RI (OR: 1.5267, 95% CI: 1.0413-2.2384, *P_IVW_*: 0.0302), IL-2 receptor α (OR: 2.3403, 95% CI: 1.0433-5.2501, *P_IVW_*: 0.0391), PECAM-1 (OR: 3.4468, 95% CI: 1.2142-9.7847, *P_IVW_*: 0.0201), Tie-2 (OR: 2.2153, 95% CI: 1.1819-4.1523, *P_IVW_*: 0.0131), and SP-D (OR: 2.3065, 95% CI: 1.1169-4.7628, *P_IVW_*: 0.0239) was associated with an increased risk of giant cell arteritis. Conversely, elevation in CEACAM1 (OR: 0.3957, 95% CI: 0.1687-0.9283, *P_IVW_*: 0.0331), MMP-1 (OR: 0.6170, 95% CI: 0.4300-0.8852, *P_IVW_*: 0.0087), NT-proBNP (OR: 0.7225, 95% CI: 0.5416-0.9639, *P_IVW_*: 0.0271), VEGFR-3 (OR: 0.6532, 95% CI: 0.4607-0.9263, *P_IVW_*: 0.0169), and angiogenin (OR: 0.0960, 95% CI: 0.0212-0.4358, *P_IVW_*: 0.0024) was associated with a decreased risk of giant cell arteritis.

### 3.4 Causal association between primary immunodeficiency-related genes and ocular diseases

We identified causal associations between 10 primary immunodeficiency-related genes and two ocular diseases (retinopathy and keratitis). Specifically, elevated levels of ST2 (OR: 1.6163, 95% CI: 1.0539-2.4788, *P_IVW_*: 0.0278) were associated with an increased risk of retinopathy. Conversely, elevated levels of cFib (OR: 0.4745, 95% CI: 0.2333-0.9651, *P_IVW_*: 0.0396), IL-2 receptor α (OR: 0.3390, 95% CI: 0.1340-0.8575, *P_IVW_*: 0.0223), and Lp(a) (OR: 0.8440, 95% CI: 0.7241-0.9836, *P_IVW_*: 0.0299) were associated with a decreased risk of retinopathy. On the other hand, elevated levels of MIP-1β (OR: 1.1117, 95% CI: 1.0395-1.1889, *P_IVW_*: 0.0020) and TARC (OR: 1.0980, 95% CI: 1.0091-1.1947, *P_IVW_*: 0.0299) were associated with an increased risk of keratitis. Conversely, elevated levels of angiogenin (OR: 0.8269, 95% CI: 0.7104-0.9624, *P_IVW_*: 0.0141), CFH (OR: 0.7197, 95% CI: 0.5198-0.9966, *P_IVW_*: 0.0476), SHBG (OR: 0.9320, 95% CI: 0.8725-0.9955, *P_IVW_*: 0.0362), and CFHR1 (OR: 0.8534, 95% CI: 0.7505-0.9705, *P_IVW_*: 0.0157) were associated with a decreased risk of keratitis.

### 3.5 Causal association between primary immunodeficiency-related genes and other varicella-zoster virus reactivation syndromes

In addition to the varicella-zoster virus reactivation syndromes mentioned earlier, we also investigated three other diseases: encephalitis, cranial nerve paralysis, and gastrointestinal infections. We found that elevated levels of eotaxin-2 (OR: 1.7531, 95% CI: 1.0824-2.8394, *P_IVW_*: 0.0225) and YKL-40 (OR: 2.1256, 95% CI: 1.0048-4.4965, *P_IVW_*: 0.0485) were causally associated with an increased risk of encephalitis, while an increase in TECK (OR: 0.6569, 95% CI: 0.4482-0.9629, *P_IVW_*: 0.0312) was associated with a decreased risk of encephalitis. Regarding cranial nerve paralysis, elevation of ErbB3 (OR: 1.1468, 95% CI: 1.0025-1.3118, *P_IVW_*: 0.0458) and IL-6R (OR: 1.2062, 95% CI: 1.0046-1.4481, *P_IVW_*: 0.0445) was linked to an increased risk, whereas an increase in IGFBP-2 (OR: 0.8035, 95% CI: 0.6802-0.9490, *P_IVW_*: 0.0100) was associated with a decreased risk. Lastly, an increase in cathepsin D (OR: 1.1303, 95% CI: 1.0152-1.2584, *P_IVW_*: 0.0254) and Apo E (OR: 1.0952, 95% CI: 1.0017-1.1974, *P_IVW_*: 0.0459) was causally associated with an increased risk of gastrointestinal infections, while an increase in haptoglobin (OR: 0.9435, 95% CI: 0.8991-0.9901, *P_IVW_*: 0.0181) was associated with a decreased risk.

### 3.6 Sensitivity analysis

In sensitivity analysis, we employed the MR-Egger intercept method to assess the presence of horizontal pleiotropy, and utilized Cochran’s Q test to quantify its heterogeneity. No evidence of horizontal pleiotropy was observed for any significant associations in the IVW test. Both the MR-Egger and IVW tests yielded Q-values indicating no significant heterogeneity for all significant associations. Additionally, the MR-PRESSO test did not identify any outliers (see **Supplementary Table** for details).

**Figure 3:**
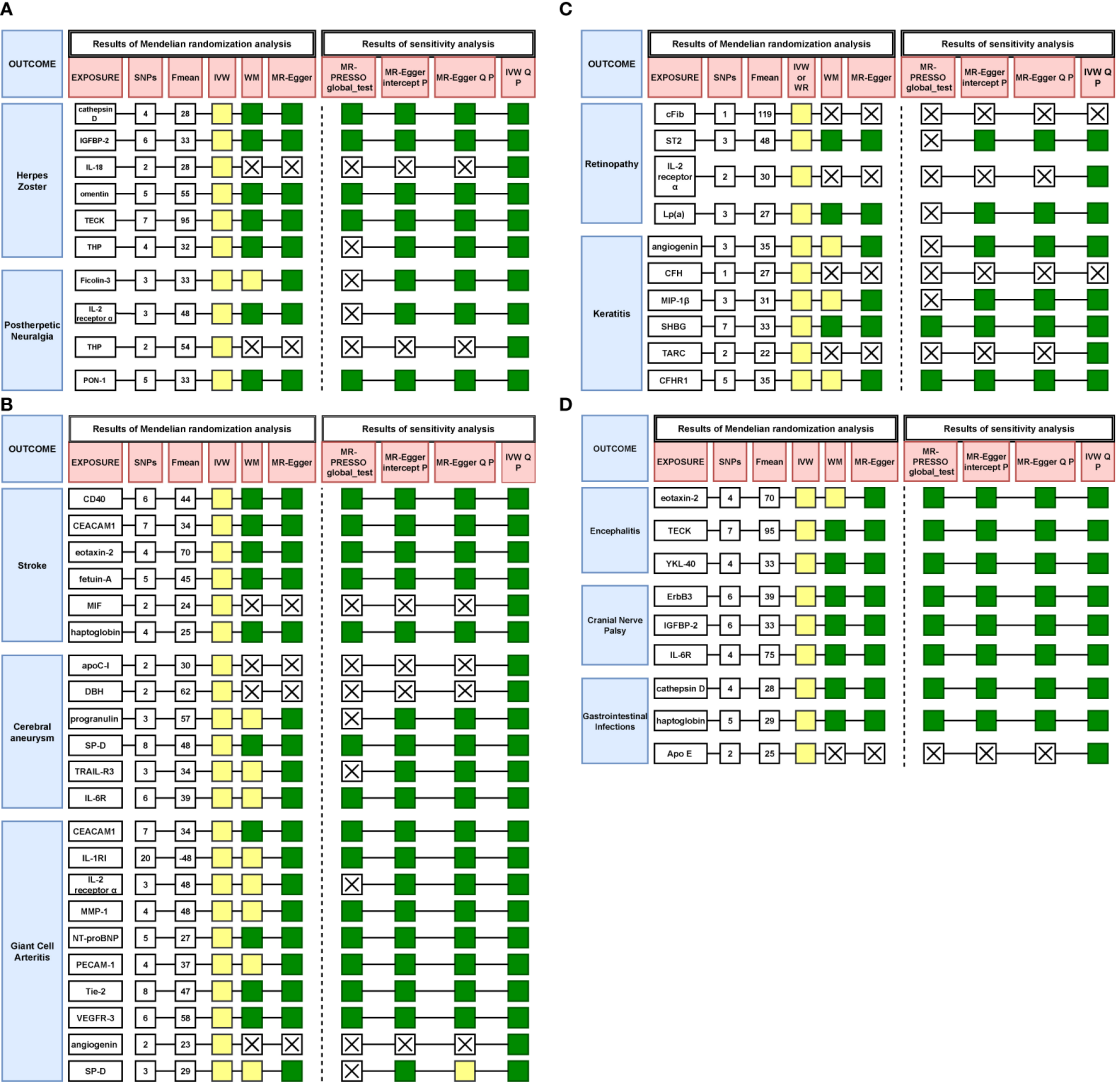
**(A)** Results of all Mendelian randomization and sensitivity analysis on herpes zoster and postherpetic neuralgia. Yellow represents *p* < 0.05, green represents *p* > 0.05, X means no result. **(B)** Results of all Mendelian randomization and sensitivity analysis on stroke, cerebral aneurysm and giant cell arteritis. Yellow represents *p* < 0.05, green represents *p* > 0.05, X means no result. **(C)** Results of all Mendelian randomization and sensitivity analysis on retinopathy and keratitis. Yellow represents *p* < 0.05, green represents *p* > 0.05, X means no result. **(D)** Results of all Mendelian randomization and sensitivity analysis on encephalitis, cranial nerve palsy and gastrointestinal infections. Yellow represents *p* < 0.05, green represents *p* > 0.05, X means no result.

In summary, we have summarized the MR results and sensitivity analysis regarding the significant associations between primary immunodeficiency-related genes and VZV reactivation syndrome, as depicted in **Figure 3**.

## 4 Discussion

NK cell deficiency (NKD) is a subtype of primary immunodeficiency disease (PID). Studies have indicated that patients with significant GATA2 gene deletions may exhibit typical infectious manifestations of NK cell deficiency, including widespread infections with VZV and severe herpes zoster (20). Additionally, research has found that congenital defects in RNA polymerase III (POL III) increase susceptibility in otherwise healthy children to severe VZV-related diseases (21). In our study, comprehensive MR analysis was conducted on GWAS data from 110 loci to explore the potential role of primary immunodeficiency-related genes in VZV reactivation syndrome.

VZV infection initially occurs as chickenpox, entering the skin nerve endings and subsequently remaining latent in the nerve ganglia. In some cases, VZV may become active again after latency, leading to the occurrence of herpes zoster. Reactivation typically occurs due to immune suppression, stress, or other triggering factors (22). Postherpetic neuralgia typically appears within about three months after the symptoms of herpes zoster have subsided. This pain stems from damage to the nerves and inflammatory reactions during the viral infection period. The pathological mechanisms of postherpetic neuralgia may involve abnormal neuronal excitability, synaptic remodeling, as well as neuronal damage and death (4). We have identified 9 primary immunodeficiency-related genes that are causally associated with both herpes zoster and its postherpetic neuralgia. These include elevated levels of cathepsin D and IL-18, which are associated with reduced risk of herpes zoster, as well as increased risk of postherpetic neuralgia with elevated levels of Ficolin-3 and IL-2 receptor α. Interleukin-18 (IL-18) is a pleiotropic cytokine secreted by activated macrophages, capable of promoting the proliferation of Th1 cells and the production of IFN-γ (23, 24). IFN-γ belongs to the type II interferon family and exerts broad immunomodulatory effects. It acts on interferon receptors on the surface of host cells, interacting with abnormal cells infected by viruses, synthesizing antiviral proteins, inhibiting intracellular viral replication, and halting viral propagation (25, 26). However, IL-18 is produced from pro-IL-18, which lacks a signal peptide and requires proteolytic processing to become active (24). Cathepsin D, as a protease predominantly found in lysosomes, plays a crucial role in protein hydrolysis processes (27, 28). Therefore, cathepsin D might be involved in the activation of IL-18, which subsequently promotes the expression of IFN-γ, thereby inhibiting the reactivation of VZV and intervening in the disease process. Ficolin-3 is a pattern recognition molecule involved in activating the lectin pathway of the complement system. Upon activation of this pathway, serum mannose-binding lectin (MBL) can bind to viral surface glycoproteins, thereby initiating downstream activation reactions and exerting antiviral effects (29, 30).Case reports have documented patients with Ficolin-3 deficiency presenting severe primary immunodeficiency, accompanied by bacterial infections and neurological complications (31, 32). This could be related to the impairment of the lectin pathway of the complement system. IL-2 receptor α serves as the specific binding receptor for IL-2, which acts as a growth factor for T lymphocytes. IL-2 binds specifically to IL-2 receptor α, thereby promoting the proliferation of T lymphocytes (33). Research indicates that patients with IL-2 receptor α deficiency are susceptible to herpesvirus, possibly due to impaired proliferation of T lymphocytes, which fail to recognize VZV (34, 35). However, there is some contradiction between these clinical findings and our study results, warranting further investigation.

After reactivation from the ganglia, VZV is transported through incoming nerve fibers to the blood vessels, leading to the infiltration of immune cells such as neutrophils and the release of activated matrix metalloproteinases (MMPs). These MMPs degrade the extracellular matrix, disrupting the internal elastic lamina and resulting in thinning of the vessel wall. Simultaneously, soluble factors are produced to promote smooth muscle cell death and accumulation of myofibroblasts within the thickened intima, leading to blood flow obstruction and eventually vascular remodeling. This cascade of events triggers various vascular disorders, including stroke, giant cell arteritis, and others (36–38). In our study, we identified 20 primary immunodeficiency-related genes associated with three vascular lesions (stroke, cerebral aneurysm, giant cell arteritis), including CD40 association with stroke, IL-6R, and SP-D association with cerebral aneurysm, and Tie-2, MMP-1 association with giant cell arteritis. For instance, CD40 is a type I transmembrane protein, belonging to the tumor necrosis factor receptor family, expressed on B cells and antigen-presenting cells, and binds to its ligand CD40L, participating in physiological processes such as thrombosis and inflammation. During viral infections, CD40 binds to CD40L, activating signaling pathways such as the phosphatidylinositol 3-kinase/Akt (PI3K/Akt) and p38 mitogen-activated protein kinase (p38 MAPK), promoting the secretion of pro-inflammatory cytokines to neutralize pathogens. However, CD40-CD40L-mediated aberrant neuroinflammatory responses may increase blood-brain barrier (BBB) permeability, promoting the formation of occlusive microthrombi (39, 40). Literature reports have shown upregulation of CD40-CD40L expression in patients with acute cerebral ischemia (41). Another study found higher levels of soluble CD40 ligand in the plasma of patients with acute ischemic stroke (42). The latest research indicates that the absence of the CD40 gene in dendritic cells or B cells can significantly reduce the incidence of autoimmune encephalomyelitis (43). Our study revealed a positive correlation between CD40 and stroke risk, providing compelling evidence for CD40 as a potential biomarker. Interleukin-6 (IL-6) is a pleiotropic cytokine with functions in regulating both the immune and nervous systems. IL-6R serves as the receptor for IL-6 and exists in membrane-bound or soluble forms in the liver and certain white blood cells. The complex of IL-6 and its receptor binds to the protein gp130, inducing its dimerization, thereby initiating intracellular signaling pathways such as JAK/STAT phosphorylation to regulate the occurrence of inflammatory responses (44, 45). Studies have shown that IL-6 can enhance macrophage infiltration in the cerebral arterial circle, promoting the rupture of estrogen-deficient-related cerebral aneurysms in mice (46). Other studies have found that treatment with anti-IL-6R antibodies can correct X-linked lymphoproliferative syndrome type 2 (XLP-2), caused by XIAP mutations, a type of primary immunodeficiency disorder (47). However, recent literature has reported that deficiency of IL-6R may lead to immunodeficiency and the occurrence of abnormal inflammatory responses (45). Our study identified a positive correlation between IL-6R and cerebral aneurysms. The discrepancy mentioned above may arise from the fact that IL-6 can bind to different forms of IL-6R, exerting both anti-inflammatory and pro-inflammatory effects. Surfactant protein D (SP-D) is an immunomodulatory agent derived from epithelial cells, belonging to the structurally related calcium-dependent C-type lectin family (48). Research on SP-D in the respiratory system is extensive, showing its roles in modulating immune balance, promoting pathogen clearance, and suppressing inflammatory responses (49). Our study revealed a causal association between elevated levels of SP-D and a decreased risk of cerebral aneurysms, providing insight into the role of SP-D in neurological disorders and vascular diseases. Additionally, we found that elevated levels of Tie-2 are associated with an increased risk of giant cell arteritis, while elevated levels of MMP-1 are associated with a decreased risk of giant cell arteritis. Recent research has shown that levels of Tie-2 are significantly higher in the blood of patients with rheumatic polymyalgia with giant cell arteritis compared to those with rheumatic polymyalgia alone and healthy controls (50). Tie-2 belongs to the tyrosine kinase receptor family and serves as the receptor for angiopoietin-1 (Ang-1). It is expressed on endothelial cells, vascular smooth muscle cells, fibroblasts, and some immune cells (51). Ang-1 binding to Tie-2 forms the Ang-1-Tie-2 signaling pathway, which can activate the p38 MAPK pathway and the PI3K/AKT pathway, exerting pro-apoptotic and anti-apoptotic effects, respectively (51–53). VZV reactivation leads to abnormal activation of the Ang-1-Tie-2 signaling pathway, promoting smooth muscle cell death and accumulation of myofibroblasts, leading to vascular remodeling. MMP-1 is a zinc-dependent endopeptidase belonging to the matrix metalloproteinase (MMPs) family (54). The MMPs family plays a significant role in central nervous system (CNS) infection inflammatory responses, especially MMP-2, MMP-3, and MMP-9 (55, 56). When VZV invades the central nervous system, triggering neuroinflammation, the MMPs family is abnormally activated. They participate in the degradation of fibronectin, laminin, and other extracellular matrix proteins, leading to the disruption of the BBB. This disrupts the normal apoptosis process of vascular cells, resulting in intimal hyperplasia and luminal narrowing, representing a series of vascular remodeling events (1, 54). Research indicates that in patients with VZV CNS infection, there is a significant elevation of MMP-3, MMP-8, MMP-9, and MMP-12 in the cerebrospinal fluid (CSF) (54). Additionally, increased levels of MMP-2 have been detected in the CSF of patients with VZV-associated vascular lesions (1). The latest research has revealed that in immunodeficient states, monocytes can produce MMP-9, mediating the infiltration of T cells into the vessel wall, leading to giant cell arteritis (57). However, studies on the role of MMP-1 in giant cell arteritis triggered by VZV reactivation are limited, and our research findings provide a different avenue for investigation.

VZV can not only reach the central nervous system through the bloodstream, triggering vascular changes and extending to the ocular arteries, leading to occlusion of the retinal arteries and causing retinal artery blockage, but it can also extend along the neural pathways within the anterior visual system, damaging the optic nerve, resulting in acute retinal necrosis (ARN) (58, 59). The latest research has found that in patients with VZV-induced ARN, there are potential pathogenic gene variations involving genes related to immunity, autophagy, and apoptosis. These variations may be associated with the pathogenesis of ARN triggered by VZV infection (60). Additionally, VZV can promote the proliferation of corneal epithelial cells and the formation of pseudodendrites, upregulate levels of pro-inflammatory cytokines and matrix metalloproteinases, leading to keratitis (61). Our study revealed the relationship between genes like ST2 and retinal lesions, as well as genes like MIP-1β and keratitis. ST2 is the receptor for interleukin-33 (IL-33) and a crucial component in IL-33 signaling, participating in biological processes such as immune responses, tissue repair, and angiogenesis through binding with IL-33 (62, 63). Research suggests that ST2 may influence the activation and migration of monocytes, regulating inflammatory responses after retinal injury to protect photoreceptors (64). Additionally, the IL-33/ST2 signaling pathway plays a role in angiogenesis, inhibiting abnormal blood vessel formation, such as suppressing retinal neovascularization in ocular diseases (65). However, in various autoimmune and inflammatory diseases, activation of the IL-33/ST2 axis can promote inflammatory responses, potentially leading to local and systemic damage (66, 67). Overall, the IL-33/ST2 axis exhibits bidirectional regulatory properties, with complex and diverse mechanisms of action. MIP-1β is a chemokine that plays a significant role in inflammatory diseases such as herpetic keratitis, caused by herpes viruses. It enhances the infiltration of white blood cells, such as neutrophils and monocytes, regulates their activation and migration, exacerbates the inflammatory response, and leads to tissue damage and disease progression (68).

The diseases of encephalitis, cranial nerve palsy, and gastrointestinal infection all involve different parts of the nervous system affected by VZV infection. Encephalitis affects the CNS, cranial nerve palsy involves the cranial nerves, and gastrointestinal infection pertains to the neurons of the gastrointestinal tract (69–71). This suggests that VZV can cause infection in various regions of the nervous system, leading to different clinical manifestations. VZV can directly infect neural tissues, triggering inflammatory responses and tissue damage, potentially resulting in neuronal dysfunction, inflammatory reactions, and tissue pathology. Encephalitis, cranial nerve palsy, and gastrointestinal infection may all arise from such direct infection and inflammatory responses. Similar to the diseases discussed earlier, these conditions may also involve latent VZV infection and reactivation. In some cases, VZV can enter a latent state post-infection, only to be reactivated under conditions of immune suppression or other influencing factors, leading to disease manifestation. Additionally, immune suppression may increase the risk of VZV-induced diseases. For instance, compromised immune systems may struggle to control viral replication and dissemination, thereby heightening the incidence of encephalitis, cranial nerve palsy, and gastrointestinal infection. Thus, while encephalitis, cranial nerve palsy, and gastrointestinal infection represent distinct diseases caused by VZV, they share common underlying mechanisms, including neural system infection, direct infection and inflammation, latent infection and reactivation, as well as the influence of immune suppression. Our research has identified nine genes that are correlated with the aforementioned three diseases. For instance, an elevation in YKL-40 levels is causally linked to an increased risk of encephalitis. YKL-40 is a cytokine-like pro-inflammatory protein produced by glial cells, typically involved in inflammation and tissue repair processes. It may participate in the pathogenesis of encephalitis through various mechanisms, including promoting inflammatory responses, influencing neuronal function, and facilitating neuroimmune reactions. Studies have shown a significant elevation in YKL-40 levels in the cerebrospinal fluid of patients with autoimmune encephalitis such as anti-γ-aminobutyric acid-B receptor encephalitis and anti-N-methyl-D-aspartate receptor encephalitis (72, 73). Furthermore, a prospective cohort study from Denmark demonstrated that VZV encephalitis predominantly occurs in elderly individuals or those with compromised immune function. The median age of patients in this study was 75 years, with approximately 39% of them experiencing immune dysfunction (74).

Our MR study has several strengths and limitations. Firstly, we conducted, for the first time, MR analysis targeting the potential causal relationship between 110 primary immunodeficiency-related genes and VZV reactivation syndrome, overcoming limitations inherent in traditional observational studies, such as reverse causation due to environmental factors and lifestyle habits. Secondly, we utilized GWAS data with a sufficient number of cases and excluded weak instrumental variables, thereby enhancing statistical power. Additionally, we performed sensitivity analyses and repeated analyses using multiple statistical models to address different pleiotropic patterns, thereby strengthening the evidence of our study findings. However, our MR study still has some limitations. Firstly, the study participants were predominantly of European descent, limiting the generalizability of the results to other populations. Secondly, we only utilized the GWAS Catalog database and the FINNGEN database, without considering other databases, which may affect the accuracy of result interpretation. Moreover, we did not explore other types of VZV reactivation syndrome and did not verify whether the causal relationship between primary immunodeficiency-related genes and other types of VZV reactivation syndrome is consistent with this MR analysis.

## 5 Conclusion

In conclusion, our MR study suggests a relationship between primary immunodeficiency-related genes and the occurrence and development of VZV reactivation syndrome. While further research is needed to elucidate the potential mechanisms of primary immunodeficiency-related genes in VZV reactivation syndrome, this study provides insights into the relationship between primary immunodeficiency-related genes and VZV reactivation syndrome, and may offer reliable and practical biomarkers for clinically predicting the risk of VZV reactivation syndrome. Further MR studies should explore the relationship between primary immunodeficiency-related genes and VZV reactivation syndrome in other databases. Additionally, expanding the study population beyond European regions will better ascertain the potential role of primary immunodeficiency-related genes in the prevention and treatment of VZV reactivation syndrome. Moreover, randomized controlled clinical trials should be conducted in the future to evaluate the intervention of specific primary immunodeficiency-related genes identified in this MR analysis in patients with VZV reactivation syndrome.

## Data Availability

The datasets presented in this study can be found in online repositories. The names of the repository/repositories and accession number(s) can be found in the article/**Supplementary Material**.
